# Transcript-based redefinition of grouped oligonucleotide probe sets using AceView: High-resolution annotation for microarrays

**DOI:** 10.1186/1471-2105-8-108

**Published:** 2007-03-29

**Authors:** Jun Lu, Joseph C Lee, Marc L Salit, Margaret C Cam

**Affiliations:** 1 Genomics Core Laboratory, National Institute of Diabetes & Digestive & Kidney Diseases, National Institutes of Health, Bethesda, MD 20892 USA; 2 Chemical Science and Technology Laboratory, National Institute of Standards and Technology, Gaithersburg, MD 20899 USA

## Abstract

**Background:**

Extracting biological information from high-density Affymetrix arrays is a multi-step process that begins with the accurate annotation of microarray probes. Shortfalls in the original Affymetrix probe annotation have been described; however, few studies have provided rigorous solutions for routine data analysis.

**Results:**

Using AceView, a comprehensive human transcript database, we have reannotated the probes by matching them to RNA transcripts instead of genes. Based on this transcript-level annotation, a new probe set definition was created in which every probe in a probe set maps to a common set of AceView gene transcripts. In addition, using artificial data sets we identified that a minimal probe set size of 4 is necessary for reliable statistical summarization. We further demonstrate that applying the new probe set definition can detect specific transcript variants contributing to differential expression and it also improves cross-platform concordance.

**Conclusion:**

We conclude that our transcript-level reannotation and redefinition of probe sets complement the original Affymetrix design. Redefinitions introduce probe sets whose sizes may not support reliable statistical summarization; therefore, we advocate using our transcript-level mapping redefinition in a secondary analysis step rather than as a replacement. Knowing which specific transcripts are differentially expressed is important to properly design probe/primer pairs for validation purposes. For convenience, we have created custom chip-description-files (CDFs) and annotation files for our new probe set definitions that are compatible with Bioconductor, Affymetrix Expression Console or third party software.

## Background

Affymetrix GeneChips™ [1,2] are widely used in biomedical research for genome-wide expression profiling. The level of gene expression is typically summarized from a probe set composed of several 25 mer probes designed to span a target region based on a UniGene cluster. Summarized expression measurements for a probe set are typically derived using a variety of algorithms, including MAS5.0 [3], model-based-expression indices (MBEI) [4], robust multi-chip-average (RMA) [5,6], and the position-dependent nearest neighbor (PDNN) algorithm [7,8].

Significant effort has been placed on extracting accurate and robust expression measurements summarized from multiple probes using a variety of statistical algorithms [9-11]. Recently, with the public release of microarray probe sequences, attention has been paid to the accuracy of individual probe annotations and its impact on gene expression data [12-17]. Probes within a probe set can be both ambiguous (non-specific, i.e. targeting multiple genes) and heterogeneous (target different transcript variants from one gene). For example, examination of the probe sequences incorporated in the Affymetrix Human Genome U95Av2 Array indicates that 10.5% of the probes are nonspecific and 9.3% are mistargeted [16]. Moreover, interpretation of probe signal is complicated by probes cross-hybridizing to similar sequences and transcript variants from alternative splicing [15,16]. It should be noted that grouping probes that map to different targets may create divergent signals that will significantly influence expression measurements from stochastic-model-based summarization approaches (*e.g*. RMA). For example, stray signal arising from probes with multiple targets within a probe set have been shown to contribute to misleading biological relationships [17]. A more nuanced approach to estimating expression levels calls for consideration of alternative splicing, as more than half of all genes are alternatively spliced in the human genome [18]. While use of the UniGene-based definition of Affymetrix probe sets may be sufficient to provide overall differential gene expression estimates, it is inadequate for distinguishing or preserving signal data arising from different transcript variants [15,19].

Several groups have explored the effects of using alternative microarray annotations. By matching probe sequences to an up-to-date Reference Sequence (RefSeq) database [20,21], Gautier *et al *[22] investigated an "alternative mapping" approach, wherein probes were grouped together if they matched a common RefSeq transcript and were excluded from a probe set if they matched 2 or more RefSeq entries. While this approach increases the specificity of each probe set, it might prove impractical in the long term with the continued growth of the RefSeq database, resulting in the erosion of probe sets over time. Carter *et al *[23] adopted a redefinition of Affymetrix probe sets where probes were matched against cDNA clones on spotted arrays. Their method showed improved concordance of expression measurements, hinting that concordant annotation would support concordance of results. In contrast, when they used the AceView transcript database to match Affymetrix probe sets containing probes that could be sequence matched to the same transcript sequence as the cDNA clone (Shared Transcript probes), they found relatively low cross-platform consistency as compared to direct sequence overlap. They postulated that the low correlation might be due to a number of factors including the presence of splice variants, the probes being subject to different cross-hybridization patterns, or incorrect clone sequence predictions [23]. More recently, Dai *et al *[13] provided a method for redefining Affymetrix probe sets using several gene and transcript databases. In their regrouping strategy, all probes that match a single transcript or gene are simply grouped into a probe set. These approaches however, did not account for the heterogeneous manner in which individual probes can target transcripts. Hence, the expression signal from a given probe set is summarized across probes that individually map to varied and/or multiple sets of transcript variants.

**Figure 1:**
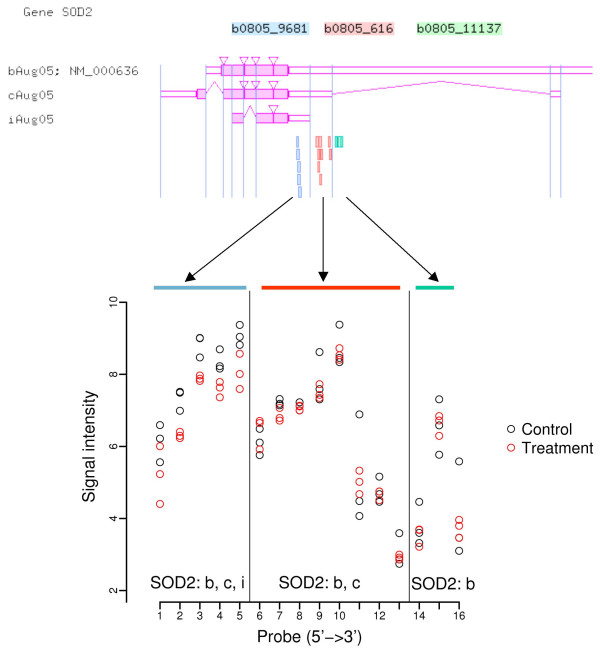
**An example of grouping Affymetrix probes into new probe sets**. The top panel shows an AceView diagram demonstrating an example of the regrouping strategy. The Affymetrix probe set "34666_at" (on GeneChip U95Av2) contains 16 probes; 5 probes forming the newly defined probe set b0805_9681 match all three transcript variants (b, c, and i) of SOD2; and 8 probes (b0805_616) match the variants b and c; and the remaining 3 probes (b0805_11137) were mapped to the variant b only. The blue vertical line indicates the exon-intron boundaries or the beginning and ends of transcripts. The bottom panel of the figure shows the log-based 2 signals in the treatment and control groups for each probe. The values from all six samples were drawn here. The probes on x-axis were ordered from 5' to 3' of the gene.

We propose here a new method for constructing probe sets for Affymetrix GeneChips based on the AceView database, a comprehensive listing of human transcripts [24,25]. The key feature of our probe set definition method is that all probes within a probe set match a common set of AceView transcripts. By doing so, transcript-level annotations are more accurate because probes regrouped within a given probe set homogeneously map to a single transcript variant or a set of alternatively spliced transcripts (see Figure 1). However, remapping probes in this way can reduce the size of a probe set, raising concerns of reliability [22,23]. Through a systematic evaluation of standard datasets, we establish the minimum probe set size required for deriving a robust expression measurement. Finally, we demonstrate the usage of our approach by reanalyzing actual gene expression data from a biological experiment and measuring the concordance between AceView transcript-matched data from two different platforms.

## Results

### Redefinition of Affymetrix probe sets

We regrouped probes into a probe set such that all probes within a probe set match a common set of transcripts, based on our current knowledge of the transcriptome found in AceView. The first step was to map individual probes to transcripts in the AceView database (see Methods). Our analysis found that the percentage of probe sequences matching to transcripts in the AceView database is very high; approximately 90% of the probes on the GeneChips U95A, U133A, and about 80% on the Human Genome U133A Plus 2.0 Array, were mapped to one or more AceView transcripts (Table 1). In contrast, only about 52% of the probes on U133 Plus 2.0 can be mapped to the RefSeq collection.

**Table 1:** Statistics of probe-to-transcript mapping and redefinition of probe sets

	**U95A**	**U133A**	**U133 Plus 2**
	
Total probe sets from Affymetrix	12,413	22,238	54,630
Total probe sets, newly defined	22,609	38,416	70,092
Identical to original Affymetrix probe sets	3,804 (17%)	5,845 (15%)	15,376 (22%)
Derived from 1 Affymetrix probe set	16,916 (75%)	27,562 (72%)	45,217 (65%)
Derived from >1 Affymetrix probe sets	1,889 (8%)	5,009 (13%)	9,499 (14%)
Probe sets containing ≥ 4 probes	13,496	19,854	42,157
Total unique probe sequences (_at probes)	199,270	241,200	593,834
Probes matching ≥ 1 transcript in AceView	180,594 (91%)	216,395 (90%)	476,624 (80%)
Probes matching ≥ 1 transcript in RefSeq	162,888 (82%)	194,728 (81%)	312,021 (52%)
Total number of AceView transcripts matched	65,952	82,457	119,204
Total number of RefSeq transcripts matched	17,370	22,787	30,788

Probes matching non-human sequences or duplicated probe sequences were excluded from the calculation.

Given a specific probe-to-transcript mapping, defining a probe set is straightforward: probes that are mapped to the same set of transcripts naturally belong to a common probe set. There are two ways in which a new probe set can be formed: it can be derived solely from a single Affymetrix probe set or it can be formed by merging probes from 2 or more Affymetrix probe sets. One example of the first scenario is shown in Figure 1 (top panel). The Affymetrix probe set 34666_at on the GeneChip U95Av2 contains 16 probes and targets the RefSeq entry NM_000636 which encodes superoxide dismutase, mitochondrial (SOD2). A "higher resolution" detailed view based on our probe-to-transcript mapping shows that this probe set actually maps to 3 AceView transcripts. Using our new probe set definition, the first five probes form one probe set since they all match transcripts SOD2.bAug05, SOD2.cAug05 and SOD2.iAug05; the next 8 probes match the transcript set SOD2.bAug05 and SOD2.cAug05, so they form another probe set, and the last 3 only match transcript SOD2.bAug05 to form yet another group. Note that, in our new probe set definition, probe sets never share probes, but the transcripts they represent may overlap.

**Figure 2:**
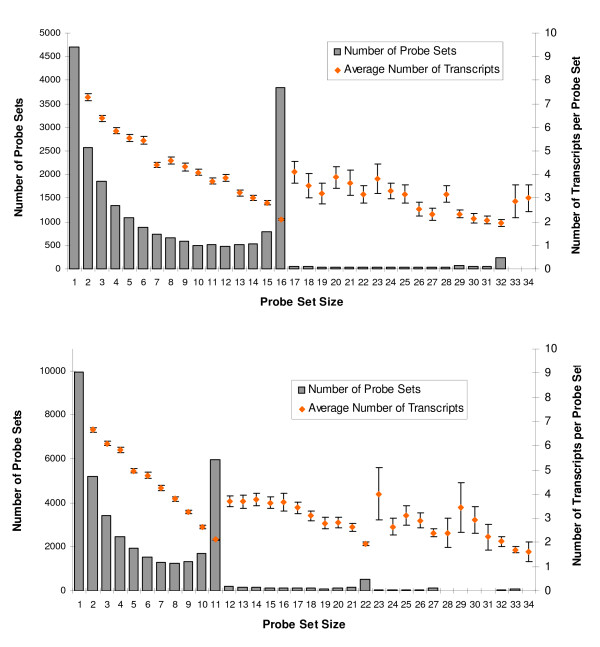
**The frequency distribution of the redefined probe set sizes for GeneChips U95A and U133A**. The number of probes in a redefined probe set is shown on the *x*-axis, and the frequency of probe sets is indicated on the left *y*-axis. The average number of transcripts (+/- SE, right *y*-axis) mapped by each probe set was also plotted against the probe set size. The upper and lower panels show U95A and U133A, respectively.

Some statistics of the newly defined probe sets for Affymetrix GeneChips U95A, U133A and U133 plus 2.0 are shown in Table 1. In the majority of cases, our redefinition splits an Affymetrix probe set into smaller probe sets; for example, only about 17%, 15% and 22% of newly defined probe sets maintain the original Affymetrix definition for the GeneChip U95A, U133A and U133 plus 2, respectively. Unlike the nominally uniform size of all original Affymetrix probe sets, such regrouping of probes results in probe sets of varying sizes. For instance, the new probe set size for the U95A chip ranges from 1 to 94 (median size, 5) and for the U133A chip, it ranges from 1 to 58 (median size, 4). The distribution of probe set sizes for U95A and U133A chips are shown in Figure 2.

Because of the large transcript-to-gene ratio in AceView, a majority of the probe sets match more than one AceView transcript. Interestingly there is an inverse relationship between probe set size and the number of transcripts a probe set targets. The inverse relationship is especially strong in probe sets derived by splitting, i.e. those probe sets smaller than the standard Affymetrix probe sets (16 probes for U95A, 11 for U133A), but it is also apparent in merged probe sets, i.e. those larger than the standard Affymetrix probe sets.

### Evaluation of the effect of probe set size on the detection of differential expression

**Figure 3:**
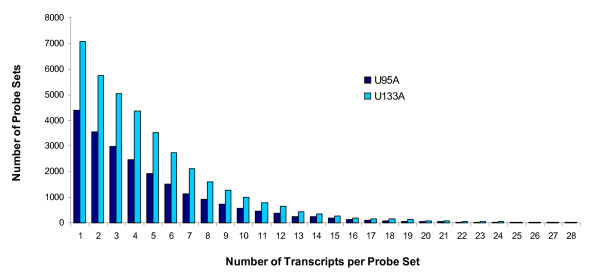
**The distribution of numbers of matching transcripts by the newly defined probe sets (U95A and U133A chip)**. The number of matching transcripts (shown on the horizontal axis) was plotted against the frequency of newly defined probe sets (y-axis). About 90% of the new probe sets match 10 transcripts or less.

We observed that increasing probe set homogeneity resulted in smaller probe sets. For example, the redefinition of probe sets on GeneChip U95A results in 20% of the probe sets containing only 1 probe and 40% having fewer than 4 probes (see Figure 3 for the probe set size distribution). To address how the probe set size influences the identification of differential expression, we created 10 artificial data sets from the U133A Latin Square data. Each data set contains expression measurements that are summarized from randomly selected subsets (1–10) of probes from each Affymetrix probe set. We chose the U133A Latin Square data because all probes of the spike-ins are known to accurately match their intended targets. By eliminating the influence of sequence specificity on gene expression measurements, the results can be evaluated solely on the basis of probe set size. All comparisons were conducted using the evaluative tools in Affycomp [11,26], and the RMA summarization algorithm was used to derive summarized gene expression values.

**Figure 4:**
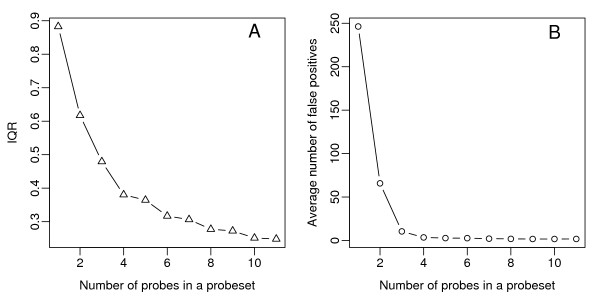
**The effects of probe set size on variability and false positive detection using summarized gene expression measurements**. These two figures are generated from data in the summarization table. The numbers of probes used for deriving the summarized expression measurements are plotted on the x-axis against (A) the IQR, used to indicate the level of variation of fold-changes (FC) of non-significant genes, and (B) the average number of false positives (called if FC>2 for non-spike-in probe sets). All data were calculated using all arrays in the U133A Latin Square spike-in data set.

**Figure 5:**
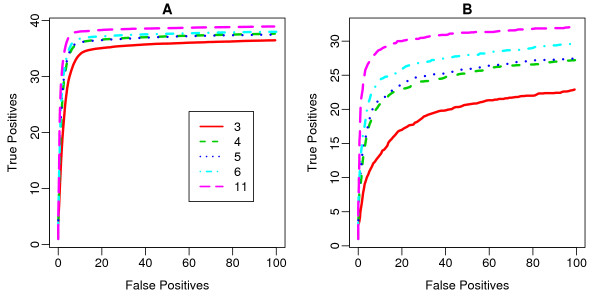
**The receiver operating characteristic (ROC) curves for expression measurements derived from various numbers of probes**. Comparison of gene expression measurements derived from 3, 4, 5, 6, and 11 probes is shown. The data summarized from 11 probes are the same as those derived from the original Affymetrix probe sets. The average ROC curves for (A) all the comparisons in the Spike-in dataset with fold changes ranging from 2 to 4092, and (B) for comparisons limited to data sets spiked-in at 2-fold.

To compare the overall variation in the data, we plotted the inter-quartile range (IQR, a measure of variance) of the log fold-changes for non-spiked-in probe sets (Figure 4A), and the average number of false positives (AFP; counted when a fold-change is > 2 for non-spiked-in probe sets; see Figure 4B) against the number of probes used for deriving the expression measurements for the probe sets. In both cases, an increased probe set size improves the reliability of expression measurements as shown by the lower variability and a significant reduction in the number of false positives (Figure 4B). Notably, there is a significant drop of both IQR and AFP when the probe set size is greater than 3. Next, to evaluate the sensitivity and specificity in detecting differential expression, Receiver Operating Characteristic (ROC) curves were plotted and compared (Figure 5). The average ROC curves for expression measurements derived from 3, 4, 5 and 11 probes (i.e. the original data) were drawn using all data where the fold-change ranges from 2 to 4096 (Figure 5A) and a subset of data where fold-change equals 2 (Figure 5B). Consistent with our earlier observations of reduced variance, at a given false positive rate, we observed increased power with increased probe set size. Importantly, the sensitivity-specificity ratio improved dramatically when the probe set size was 4 or more.

### Application of new probe set definition to biological datasets

By regrouping probes into homogeneous probe sets using the AceView mapping, gene expression is examined at the transcript level rather than the gene level. Higher resolution probe set definitions allowed us to identify specific transcript variants that were initially undetectable within the original heterogeneous probe sets. In an earlier experiment using the Affymetrix platform, we compared pancreatic tumor cells prior to and after serum removal to study early events accompanying islet cell differentiation [27]. Here, this data set was reanalyzed with both the original and the new probe set definitions and the two lists of differentially expressed genes were compared. In this analysis, we consider a gene or a probe set differentially expressed if the false discovery rate (FDR) adjusted p-value is less than 0.05 and the fold-change (up or down-regulated) is greater than 1.7.

**Figure 6:**
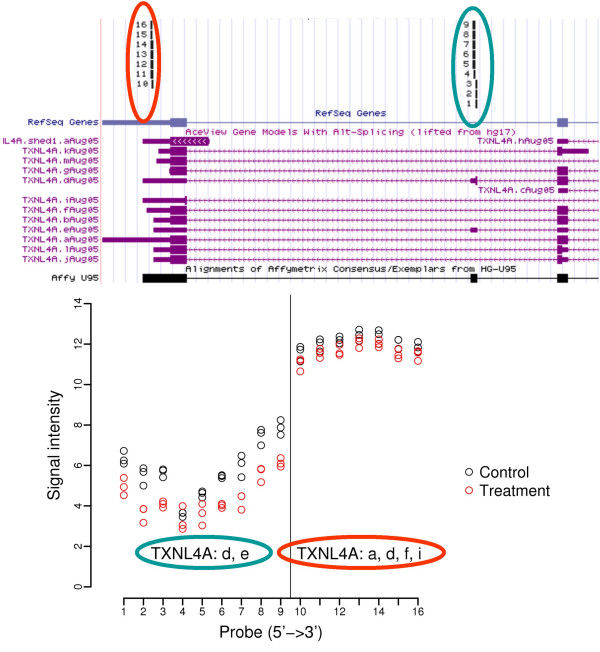
**The interrogation of specific transcripts using the redefined probe sets**. The top panel shows the annotation of Affymetrix probes to AceVew transcripts, drawn by BLAT [44]. The Affymetrix probe set "33631_at" (on GeneChip U95Av2) contains 16 probes; 9 probes match two transcript variants (d and e) of TXNL4A, forming a new probe set by our definition (circled in blue); and the remaining 7 probes match the variants a, d, f and I, forming another new probe set (circled in red). The bottom panel of the figure shows the log-based 2 signals for each probe in the treatment and control groups (3 samples in each group). The probes on x-axis were ordered from 5' to 3' of the gene. Notice that the expression values are relatively homogeneous within each new probe set (separated by the vertical line, and the target transcripts were also circled in blue or red). The expression level differences between two groups are most clearly seen in the group on the left side (circled in blue).

First, we observe that a vast majority of genes were identified by both probe set definitions. Of the 425 Affymetrix probe sets shown to be differentially expressed, 367 (86%) represent the same genes as those identified using the new probe set definition. Although the gene lists derived from both probe set definitions largely overlap, the new probe set provides additional detailed information on which set of transcripts are likely differentially expressed. One example is shown in Figure 6. The Affymetrix probe set "33631_at" is significantly differentially expressed (FDR adjusted p-value = 0.02), and it targets the gene TXNL4A which has at least 6 transcript variants (see the top-panel). Our new definition divides this probe set into two new probe sets, one of which (circled in blue in Figure 6) specifically targets transcripts d and e and seems to be highly significant (FDR adjusted p-value < 0.01). The other probe set (circled in red) targets potentially many different transcript variants at the 3' end of the gene and does not show significant difference in expression levels between control and treatment samples (FDR adjusted p-value = 0.23). In this example, applying the new probe set definition clearly helps to identify the specific transcript variants that are likely differentially expressed. It should be noted that such transcript-level information is critical for designing probes and primers for Real-Time PCR (RT-PCR) validation. For example, selecting the primers/probes targeting the 3' end of TXNL4A as shown in Figure 6 would mask the detection of expression level differences between the treatment and control group. Table 2 lists genes which were identified as differentially expressed by both the Affymetrix and the new probe set definitions, but the new sets increased the resolution of the assay, narrowing the detection of differential expression changes to a specific set of transcripts,

**Table 2:** Increased transcript specificity by using the newly defined probe sets

	**Affymetrix Probe Sets**				**Redefined Probe Sets**		
Identifier	Fold	Gene symbol	No. of variants	Identifier	Fold	No. of variants	Transcripts
38010_at	2.2	BNIP3	3	b0805_15050	2.24	2	BNIP3: a, b
31852_at	2.06	ANKMY2	3	b0805_979	2.01	2	ANKMY2: a, b
36813_at	-3.15	TRIP13	3	b0805_19653	-3.94	2	TRIP13: a, e
38821_at	1.89	PGRMC2	3	b0805_3475	1.75	2	PGRMC2: a, d
33631_at	-1.78	TXNL4A	5	b0805_2958	-2.71	2	TXNL4A: d, e
40916_at	1.97	BEXL1	3	b0805_642	2.35	2	BEXL1: a, c
39109_at	-2.49	TPX2	4	b0805_8744	-2.44	2	TPX2: a, b
37985_at	-1.78	LMNB1	3	b0805_7685	-1.75	2	LMNB1: a, c
34363_at	2.33	SEPP1	3	b0805_18077	6.87	2	SEPP1: c, f
37582_at	2.06	KRT15	4	b0805_20129	2.04	2	KRT15: a, c
34805_at	-2.09	MGC2574	3	b0805_16254	-1.9	2	MGC2574: a, c
40117_at	-2.26	MCM6	2	b0805_21229	-2.38	1	MCM6: a
37005_at	2.35	NBL1	3	b0805_14295	2.34	2	NBL1: a, b
40710_at	1.78	CLGN	2	b0805_21744	1.9	1	CLGN: b
36591_at	-1.71	TUBA1	7	b0805_9382	-1.98	1	TUBA1: a
41583_at	-2.98	FEN1	3	b0805_6644	-2.79	2	FEN1: a, c
35367_at	1.73	LGALS3	8	b0805_18811	1.78	2	LGALS3: a, c
41274_at	1.7	DKFZp667G2110	2	b0805_4597	1.74	1	DKFZp667G2110: a
39329_at	-1.95	ACTN1	4	b0805_9591	-2.03	2	ACTN1: a, c
39809_at	2.19	HBP1	3	b0805_22136	2.17	2	HBP1: a, b
33891_at	2.77	CLIC4	2	b0805_15863	2.76	1	CLIC4: b
39441_at	2.92	LANCL1	2	b0805_1092	3	1	LANCL1: a
1884_s_at	-2.12	PCNA	5	b0805_5909	-2.04	2	PCNA: a, b
41747_s_at	2.67	MEF2A	2	b0805_21100	3.22	1	MEF2A: e
35767_at	1.82	GABARAPL2	3	b0805_5736	2.01	2	GABARAPL2: a, c
41415_at	-1.85	BYSL	3	b0805_3838	-2.07	2	BYSL: a, b
40414_at	-2.44	VARS	4	b0805_873	-2.34	2	VARS: a, g
38728_at	-2.06	NUP205	4	b0805_14227	-2.36	1	NUP205: a
39385_at	-2.6	ANPEP	3	b0805_3943	-2.59	1	ANPEP: a

The probe set identifiers ending with "_at" are from Affymetrix; and those beginning with "b0805_" are newly defined here. Fold changes are ratios of the mean values of the treatment versus values of the control group, and the negative values indicate changes in the opposite direction.

Comparing the two gene lists also identifies about 13–16% of genes/probe sets which can only be identified as being differentially expressed with either the original or the new probe set definition. The majority of the genes missed by either definition have p-values and fold-changes close to the threshold chosen above (data not shown), suggesting that the results using the two probe set definitions are largely similar. However, in a few cases, we observed that with higher resolution of the new probe set definition, new transcript level changes are also uncovered. One example is shown in Figure 1, where the probe-level signals were plotted (bottom panel). The original Affymetrix probe set "34666_at" is not considered to be differentially expressed at the 5% significance level (FDR adjusted p-value = 0.44). However, in our new AceView-based definition, 16 probes in this probe set (shown in Figure 1) were divided into 3 new probe sets. One probe set (b0805_9681) which maps to transcript variants b, c, and i of SOD2, appears to be significantly downregulated (FDR adjusted p-value = 0.04, fold-change = 1.9); the other two new probe sets map to variants b and c (b0805_616) or to b (b0805_11137) only, and both probe sets are not significantly changed (data not shown). From these results, we can infer that variant i might be significantly differentially expressed since it is being uniquely interrogated by b0805_9681. Another example is the Affymetrix probe set "37513_at" representing Stearoyl-CoA desaturase (SCD). This gene was found to be differentially expressed only by using the new definition (FDR adjusted p-value = 0.02, fold-change = 2.3), and this change was validated by real-time PCR (2.5-fold, p < 0.05).

### Comparing cross-platform data using the new probe set definition

To further evaluate our probe set redefinition, we conducted a full-scale transcript-level cross-platform comparison. Most previous cross-platform analyses have utilized gene-level annotations. We hypothesized that transcript-level annotation of probe sets can be used to better match probe identifiers across different microarray platforms, thereby improving the consistency of cross-platform measurements. Hence, two identifiers (one on each platform) are matched if both map to a common set of AceView transcripts, whereas the original Affymetrix probe sets were matched to Codelink probes using common UniGene identifiers (see Methods for details). Table 3 shows the cross-platform concordance measured by the Pearson's correlation of log-ratios. The overall correlation coefficients calculated using the UniGene-based matches are about the same as those using the transcript-based matches. However, after excluding the less reliable probe sets (i.e. probe sets with fewer than 4 probes), the correlation derived from transcript-based matches is higher across three replicates (p = 0.03). The consistent, though small, improvement in correlations suggests that there may be better cross-platform comparability when the transcriptome is probed with higherresolution. Similar improvements are observed when using RefSeq-based mapping by Dai et al [13]. Furthermore, we observed poor correlation between the Affymetrix and CodeLink data for newly defined Affymetrix probe sets with only 1, 2 or 3 probes, providing additional evidence that measurements of newly defined probe sets with small probe set sizes (e.g. less than 4 probes) are less reliable than those with larger probe set sizes.

**Table 3:** Pearson's correlations between Affymetrix and Codelink data

Cross-platform Comparison		Pearson's correlations		
		
	*N*	D1/C1	D2/C2	D3/C3
UniGene-based match	7,399	0.55	0.55	0.58
AceView-based match	4,064	0.55	0.57	0.61
Probe sets with size ≥ 4	3,694	0.59	0.61	0.63
Probe sets with size < 4	370	0.34	0.37	0.39
RefSeq-based match*	7,801	0.60	0.61	0.64

The D1-D3 and C1-C3 are replicates in the treatment group and the control group respectively. The correlation is calculated on log-ratios, where the pairing of technical replicates is arbitrary. The best concordance between two platforms was observed using AceView-based transcript-level probe sets (with size ≥ 4) and RefSeq based probe sets.

*Using RefSeq-based mappings by Dai et al [13]

## Discussion and conclusion

In this report we present a new approach to integrating an up-to-date probe annotation into routine Affymetrix array analysis. Although the Affymetrix GeneChip arrays are not particularly designed to detect alternative transcripts, with careful transcript-level annotation we have demonstrated that specificity can be achieved by using the new probe set definition. One of the advantages of using the newly redefined probe sets is that it allows the examination of gene expression in-depth at the transcript level, providing a level of clarity in data interpretation unavailable at the gene level or even at the RefSeq transcript level. With the total number of AceView transcripts at 243,707 compared with 39,115 in RefSeq, probes from all chips examined matched approximately four times the number of transcripts in AceView relative to ones annotated in RefSeq. In addition, ~80% of all U133 Plus 2.0 array probes matched AceView transcripts, which was ~50% more than the number that matched to RefSeq. Such a detailed view is necessary if one needs to design primers or probes for quantitative-PCR verification. Moreover, our method naturally separates the ambiguous and cross-hybridizing probes and automatically groups gene specific probes.

Although our approach to grouping probes into probe sets is independent of the particular transcript database being used, we consider AceView to be the most comprehensive and accurate database publicly available for conducting such transcript-level reannotation of probes. In comparison to RefSeq, which is a highly curated yet incomplete mapping of the transcriptome, AceView annotations identify on average, 5.0 transcripts per gene, greatly exceeding that of RefSeq's 1.3 per gene. Furthermore, in annotating the ENCODE region[28], the quality of AceView transcript annotation has been shown to be comparable with the gold-standard manual Havana annotation. If the overall depth and quality was considered, among the 16 annotation approaches compared, AceView is "by far the closest match" to the painstaking manual transcript annotation [24].

As a result of maintaining homogenous probe sets and excluding ambiguous and cross-hybridizing probes, this new redefinition often results in small probe sets (i.e. having fewer than 4 probes). Using a random sampling of probes from the original Affymetrix probe sets, we demonstrate that, without considering the annotation issue, at least 4 probes may be required for deriving reliable expression measurements. From all the arrays studied, these adequately sized probe sets comprise 58% of all new probe sets. Our observation that probe sets with fewer than 4 probes yield poor data may arise from a number of factors. Non-functioning probes may exist for certain probe sets: for instance, on the U95A chip, a number of probe pairs for probe sets 407_at and 36889_at were found to perform poorly [29]. Deviation of probe length on the array from the designed 25-mer, due to synthesis inefficiency, may also contribute to both variability and poor probe performance, including array-to-array variation [30]. Non-functioning probes due to the latter case are particularly difficult to trace and this problem is probably only circumvented by integrating data from multiple probes.

A recent paper by Dai *et al *[13] provided a method for redefining Affymetrix probe sets using several gene and transcript databases. Their regrouping strategy, however, is fundamentally different from the current method in that with their method, all probes that match a single transcript or gene are simply grouped into a probe set. However, their method does not generate "transcript-specific" probe sets for genes with multiple transcripts, and does not eliminate probe sets with multiple targets [17]. Hence, there may be some probes within a newly regrouped probe set that may actually cross-hybridize to a different transcript. An example of this can be considered using Figure 1 to demonstrate. According to their method, transcript b (NM_000636) would utilize all probes from the original Affymetrix probe set. With our redefinition, only the last 3 probes (b0805_11137) are specific for this transcript. Furthermore, with their method, transcript c of SOD2 will be represented by merging our newly redefined probe sets, b0805_9681 and b0805_616. It is clear that the probes from these different probe sets show gene expression profiles that are markedly different. Thus, we expect that the specificity and homogeneity within our probe sets will result in more accurate gene expression measurements, as recently suggested in [17]. To demonstrate, using the RefSeq-based remapping of Dai et al, there were clear differences in relative gene expression changes obtained, examples of which are presented in Supplemental Figures 1 (SOD2) and 2 (TXNL4A) [See Additional file 1]. However, while these examples demonstrate differences in individual results, they did not translate into global improvements in the cross-platform correlation using our current method over RefSeq-mapped probe sets. A possible probe selection bias towards abundant transcripts through the use of RefSeq-based probe sets may account for this lack of difference.

The quality of the new probe set definition depends on a number of factors. It is notable that 2–4% of probes on the human arrays studied are ambiguous (i.e. they align to multiple genes), and the resulting probe sets should be used with caution. The gene(s) targeted by each new probe set are made available in the annotation files downloadable from [35]. In addition, because of the relative lack of information on poly-A sites, it should be stressed that the current probe sets may not accurately reflect the regulation that occurs at the level of alternative poly-adenylation. For instance, regrouping of probes derived from more than one Affymetrix probe set may have resulted from poly-A sites currently unannotated in AceView. Conversely, there may have been some probe sets which are split by the presence of partial cDNAs in AceView that do not clearly define a poly-A site. As greater sequence coverage and refinement of the human genome become available, a strategy such as described here would permit continuous updating and refinement of probe sets, and better interpretation of results, based on the latest knowledge [15]. While we used AceView for redefining probe sets, the method of regrouping probes can be applied using any public or "in-house" database, and the guidelines provided here for creating a viable "probe set" should be generally applicable. This method is also particularly relevant with the recently developed exon arrays which have genome-wide probe content specific to individual exons, observed or predicted. A method to estimate quantitative expression data at the gene-level is suggested in [31]. This approach employs a variety of annotations for grouping probes into sets, followed by summarization with the PLIER algorithm [32] or a derivative of it. However, we note that while transcript level annotations can be derived from naturally homogeneous exon-level probe sets, preliminary examination indicates that not all probe sets are actually homogeneous. Exon array probes are based on probe selection regions, or PSR, which are built around "exon clusters" or overlapping exons that may or may not share similar splice sites [33]. Hence exon arrays, while providing a significant improvement over 3' expression arrays towards transcript specificity, may continue to heterogeneously target multiple transcript variants. Since an array design of 4 probes per single exon minimally satisfies the requirements for a summarized expression value, splitting these into smaller sets might further degrade the accuracy of these probe sets. With the rising number of alternative variants annotated in AceView and elsewhere, transcript-specific arrays would require much higher densities to achieve even greater resolution while maintaining an adequate number of probes from which to extract accurate expression data. As such, probes on whole genome tiling arrays designed for transcript mapping could be grouped *de novo *based on AceView transcripts and are a viable platform for this strategy.

In conclusion, our transcript-level reannotation and redefinition of probe sets complement the original Affymetrix design. Redefinitions introduce probe sets whose sizes may not support reliable statistical summarization; therefore, we advocate using our transcript-level mapping redefinition in a secondary analysis step rather than as a replacement. Knowing which specific transcripts are differentially expressed is important to properly design probe/primer pairs for validation purposes. The custom chip-description-files (CDFs) and annotation files for our new probe set definitions [35] are compatible with Bioconductor, with Affymetrix's Expression Console or third party software.

## Methods

### Probe-to-transcript mapping and the redefinition of probe sets

We regrouped probes into probe sets based on AceView, a comprehensive human transcript annotation database [25]. The AceView transcripts are reconstructed from mRNAs in three databases: GenBank, dbEST and RefSeq; therefore, AceView shows a broader coverage and identifies many more transcript variants than RefSeq alone [24]. Affymetrix probe sequences for the various types of GeneChips were downloaded from [34]. Each probe sequence was then matched against transcripts in AceView (Release August 2005; human 35.4/hg17; non-cloud genes). Here we named a probe by its Affymetrix probe set identifier and the interrogation position (seen in downloaded probe sequence files) separated by '-'. A probe is considered to match a transcript if the probe shares 22 or more contiguous base pairs (bps) with that transcript sequence. The length cutoff of 22 was chosen based on our empirical observation that in the Affymetrix U95A spike-in dataset (available at [29]), probes matching 22 bases of a transcript are capable of detecting 2-fold differences (data not shown). Through this mapping procedure, we constructed a hash table where the keys and values are probe sequences and sets of AceView transcript identifiers, respectively. Next, probes are grouped into a probe set if they all match exactly the same set of transcripts (as shown in Figure 1). If a probe does not share transcript mapping with any other probe, it is assigned as an independent probe set. The naming of newly defined probe sets is somewhat arbitrary. A set of tab-delimited files containing the annotation of newly defined probe sets, including probe set names, the original Affymetrix probe set definition, gene symbol(s) and description, are available for download at [35]. The chip description files (CDFs) required for mapping the probe positions on the chips to the sequence annotation were made using the R package "altcdfenvs" [22,36], These CDF files and corresponding CDF packages are compatible with other bioconductor packages, such as "affy", to derive expression summary values for the newly defined probe sets, and are available for download as well [35]. In addition, custom CDF files which are compatible with third party software are also available for download.

### Performance testing using size-based definition of Affymetrix probe sets

To evaluate how many probes in a probe set are required to derive a robust expression measurement, we ran a simulation to test the accuracy and consistency of a standard data set where probe sets are redefined based on having different sizes (i.e. having different numbers of probes). To do this, all probe sets for the U133A Genechip were artificially redefined by size (denoted as d1, d2, ..., d10), by randomly sampling various numbers of probes from the original probe sets. For example, the original Affymetrix probe set on GeneChip U133A has 11 probes; however, in our artificial probe set definition, say d2, each probe set only contains 2 probes which are randomly drawn from the corresponding original Affymetrix probe sets. Next, using the R package altcdfenvs, we built 10 chip design files (CDF), with each corresponding to a probe set size-based definition.

Using these CDF files and standard summarization approach RMA [6], we generated 10 artificial data sets from the original U133A Spike-In data sets downloaded from the Affymetrix website [29]. The "affy" package in bioconductor was used to read and process the raw.cel files. To make comparisons consistent, the array preprocessing is the same for all simulated datasets, using the CDF file from Affymetrix (the default in affy). We chose RMA background correction and quantile normalization [6]. The normalized probe-level expression data was then used for deriving gene expression summary values. Since a set of standard evaluation tools are available in Bioconductor's "affycomp" package [9,11] for generating a series of comparison plots and summarization tables, we used it to compare the gene expression summaries derived from different-sized probe sets. The 10 sets of expression measurements from the simulation study are available for download at our website [35].

### A cross-platform comparison and analysis of a biological data set

The expression data for Affymetrix and Codelink were obtained as described in [27]. In the cross-platform comparison, we compared RNAs from 6 samples: 3 technical replicates from PANC-1 cells grown in serum-rich medium (the control group) and 3 replicates from cells one day after the serum was removed (the treatment group). Identical RNA samples were applied to the Affymetrix U95Av.2 arrays and the Codelink UniSet Human I Bioarrays from Amersham (30 mer oligonucleotide probes). The raw expression data from both platforms are available at [37]. For the Affymetrix platform, data were pre-processed and normalized using the RMA method available in the bioconductor "affy" package [38]. For the Codelink data we used quantile normalization as used in RMA, and only probes with measurements labeled as "Good" across all 6 samples were included in our analysis. Next, the three individual log_2 _ratios of expression values for the treatment versus control samples were calculated, where the pairing of a sample in the control group with a sample in the treatment group is arbitrary. These log-ratios were used as recommended [39] to calculate and compare the Pearson's correlations for data from the two platforms. The probe identifiers from the two platforms were cross-mapped by two methods: the UniGene IDs [40] and the AceView transcripts. First, RESOURCERER [41] (version July 2005) was used to carry out the UniGene-based mapping between the Codelink identifiers and the original Affymetrix probe sets. The AceView-based mapping is straightforward: a Codelink probe is considered matching a newly defined Affymetrix probe set if both are mapped to the same set of AceView transcripts.

We conducted a comparison between two groups (cells with serum versus cells with one-day after serum removal) using our newly defined probe sets and the original Affymetrix probe sets. For simplicity we averaged data from 3 technical replicates into one biological replicate in each group (so each group contains three biological replicates). For the new probe set definition, probe sets with 3 or less probes were excluded from the analysis. The empirical Bayes method [42] was applied to calculate t-statistics and p-values and the p-values were further adjusted by the False Discovery Rate (FDR) approach using the "p.adjust" function in the "limma" package [43].

## Authors' contributions

JL and MCC developed the method. JL carried out the simulation and data analysis. MCC supervised the study and assisted with the data interpretation. All authors contributed to the writing, read and approved the final manuscript.

## Supplementary Material

Additional file 1 Gene expression changes measured by regrouping of probe sets 34666_at (SOD2), and probe sets 33631_at and 33632_g_at (TXNL4A) using our (AceView) method compared with the regrouping method of Dai et al [13] against RefSeq. Click here for file
