# Supplementary material for: Transcript-based redefinition of grouped oligonucleotide probe sets using AceView: High-resolution annotation for microarrays
Source: BMC Bioinformatics. 2007 Mar 29;8:108. doi: 10.1186/1471-2105-8-108 (PMC1853115; doi:10.1186/1471-2105-8-108)
Supplement: Additional file 1 — Gene expression changes measured by regrouping of probe sets 34666_at (SOD2), and probe sets 33631_at and 33632_g_at (TXNL4A) using our (AceView) method compared with the regrouping method of Dai et al [13] against RefSeq. [file 1471-2105-8-108-S1.doc]

Supp. Fig. 1. Gene expression changes measured by regrouping of probe set 34666_at (SOD2) using our (AceView) method and of Dai et al (13) against RefSeq:


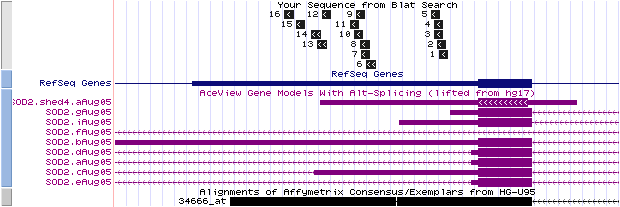


| Probes | From | AceView Probe Sets | Fold Change | Transcripts |
| --- | --- | --- | --- | --- |
| 1-5 | 34666_at | b0805_9681 | -1.87* | b,c,i |
| 6-13 | 34666_at | b0805_616 | -1.06 | b,c |
| 14-16 | 34666_at | b0805_11137 | -1.04 | b |
|  |  |  |  |  |
|  |  | RefSeq Probe Set^+^ |  |  |
| 1-16 | 34666_at | NM_000636_at | -1.29 | NM_000636 |

*p<0.01

^+^Using Dai et al (13)

Supp Fig. 2. Gene expression changes measured by regrouping of probe sets 33631_at and 33632_g_at (TXNL4A) using:

(A) AceView Transcripts by current redefinition strategy


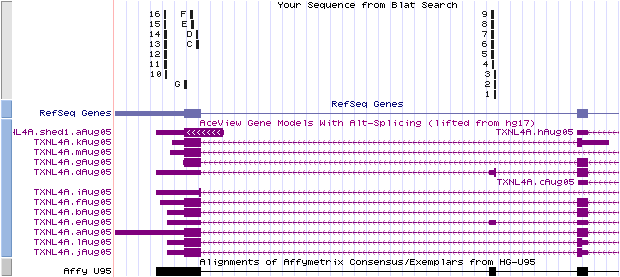


| Probes | From | AceView Probe Set | Fold Change | Transcripts |
| --- | --- | --- | --- | --- |
| 1-9 | 33631_at | b0805_2958 | -2.71* | d,e |
| 10-16 | 33631_at | b0805_2537 | -1.35 | a,d,f,i |
| C-G | 33632_g_at | b0805_8290 | 1.01 | a,b,d,e,f,g,i,j,k,l,m |

*p<0.01

(B) RefSeq Transcripts using redefinition strategy by Dai et al (ref. 13).


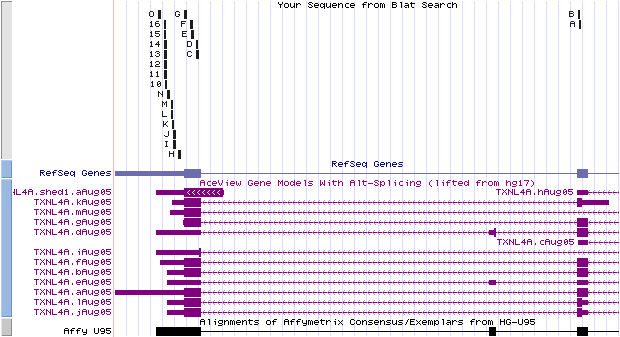


| Probes | From | Probe Set | Fold Change | Transcripts |
| --- | --- | --- | --- | --- |
| 10-16 | 33631_at | NM_006701_at | -1.17 | NM_006701 |
| A-N, O | 33632_g_at |
